# Efficient AES Side-Channel Attacks Based on Residual Mamba Enhanced CNN

**DOI:** 10.3390/e27080853

**Published:** 2025-08-11

**Authors:** Zhaobin Li, Chenchong Du, Xiaoyi Duan

**Affiliations:** Beijing Electronic Science and Technology Institute, Beijing 100070, China

**Keywords:** side-channel attack, AES, deep learning, Residual Mamba, CNN, guessing entropy

## Abstract

With the continuous advancement of side-channel attacks (SCA), deep learning-based methods have emerged as a prominent research focus due to their powerful feature extraction and nonlinear modeling capabilities. Traditional convolutional neural networks (CNNs) excel at capturing local temporal dependencies but struggle to model long-range sequential information effectively, limiting attack efficiency and generalization. In this paper, we propose a hybrid deep neural network architecture that integrates Residual Mamba blocks with multi-layer perceptrons (MLP) to enhance the modeling of side-channel information from AES implementations. The Residual Mamba module leverages state-space modeling to capture long-range dependencies, improving the model’s global temporal perception, while the MLP module further fuses high-dimensional features. Experiments conducted on the publicly available ASCAD dataset targeting the second byte of AES demonstrate that our model achieves guessing entropy (GE) rank 1 with fewer than 100 attack traces, significantly outperforming traditional CNNs and recent Transformer-based models. The proposed approach exhibits fast convergence and high attack efficiency, offering an effective new paradigm for deep learning in side-channel analysis with important theoretical and practical implications.

## 1. Introduction

In contemporary cryptography, the Advanced Encryption Standard (AES) is widely applied in communication, secure payment, IoT devices, and many other fields. Thanks to its solid mathematical foundation and standardized implementation, AES theoretically provides extremely high security against brute-force attacks. However, in practical applications, encryption operations may leak information through physical signals such as power consumption, electromagnetic radiation, and timing, providing attackers with additional analysis pathways. These types of attacks are collectively referred to as side-channel attacks.

Side-channel attacks do not directly target the cryptographic algorithm itself, but rather analyze the power consumption or electromagnetic wave traces generated by encryption devices during key processing to restore internal states or even recover keys. Since the advent of power analysis attacks, side-channel analysis has gradually become an important field in modern cryptographic engineering research. Among these, deep learning-based side-channel attacks (DL-SCA) have become a mainstream research hotspot in recent years, particularly showing great potential in processing large-scale leakage data and modeling nonlinear information leakage. Although the Mamba architecture, as a novel deep learning architecture based on state space models, has attracted widespread attention since its release in late 2023 [1,2], its application in the field of side-channel analysis (SCA) remains unexplored. Through comprehensive investigation of existing literature, we made the following discoveries.

Current Status of Deep Learning Methods in SCA: Current deep learning methods in SCA tasks primarily include traditional architectures such as multi-layer perceptrons (MLPs) and convolutional neural networks (CNNs) [3,4], as well as recently emerged diffusion model-based data augmentation approaches [5]. While these methods perform well in certain scenarios, they face challenges in computational complexity and memory consumption when processing long power trace sequences.

Application Gap of Mamba Architecture in Related Fields: Although the Mamba architecture has demonstrated excellent performance across multiple domains including natural language processing, computer vision, and genomics [1,6], no related application research has been found in the field of cryptographic security analysis, particularly in side-channel analysis. Current literature search results indicate that research work applying the Mamba architecture and its variants (such as Residual Mamba modules) to SCA tasks remains unexplored.

Necessity of Technological Innovation: SCA tasks typically require processing power trace sequences containing thousands of time points. Traditional attention mechanism-based methods face quadratic complexity computational bottlenecks when handling such long sequences [7]. The Mamba architecture, with its linear time complexity and selective state space model advantages, provides a new technical pathway to address this challenge.

Therefore, this research represents the first introduction of Residual Mamba modules into SCA tasks, filling the technical gap in this interdisciplinary field and providing a new architectural choice for improving the efficiency and accuracy of side-channel attacks.

Traditional DL-SCA methods are mostly based on convolutional neural networks (CNNs), such as the shallow CNN structure used by the ASCAD dataset proposers, which has been proven capable of recovering key bytes without prior leakage model conditions. However, these models typically excel at local modeling but have limitations in handling global temporal dependencies. As the number of model layers increases, although expressiveness is enhanced, computational complexity rises sharply, while also facing challenges such as overfitting and training instability.

To further improve model performance in side-channel analysis tasks, recent studies have attempted to introduce new structures such as Transformers, self-attention mechanisms, and state-space modeling. In particular, the Mamba architecture proposed in 2023, as a linear time complexity state-space sequence modeling method, has achieved comparable or even superior results to Transformers in long sequence modeling tasks (such as speech, finance, and remote sensing).

Based on this foundation, this paper proposes a novel deep neural network structure that integrates the local feature extraction capability of traditional CNNs with the global temporal modeling capability of Residual Mamba modules, combined with multi-layer perceptrons (MLPs) for high-dimensional feature fusion, forming a hybrid neural network architecture suitable for efficient AES side-channel attacks.

This architecture has the following advantages:

- High structural innovation, being the first to introduce Residual Mamba modules in SCA tasks, significantly improving the modeling capability for temporal dependency information;
- High attack efficiency, achieving GE = 1 with fewer than 100 attack traces and within 25 training epochs on the ASCAD dataset;
- Strong model stability, integrating residual connections and regularization mechanisms to make the training process more stable with stronger generalization capability.

The goal of this research is to verify the feasibility and effectiveness of integrating Mamba and CNN structures in SCA scenarios and compare them with traditional methods. The final results show that this method has significant advantages in training efficiency, GE convergence speed, and attack performance, providing a reference path for designing efficient attack models on low-resource devices.

## 2. Related Work

In recent years, the application of deep learning in side-channel attacks has achieved breakthrough progress, completely changing the paradigm of traditional cryptographic analysis. Before the rise of deep learning, side-channel attacks mainly relied on traditional methods such as Template Attacks and Differential Power Analysis (DPA) [4], which required extensive manual feature engineering and domain expertise, with limited robustness to noise and countermeasures.

Early research mainly focused on convolutional neural network structures. Prouff et al. first proposed using deep CNN architectures on the ASCAD dataset in 2018, achieving efficient recovery of AES key bytes without relying on preprocessing (such as key point selection or dimensionality reduction) [4]. Their CNN model included multiple convolutional and pooling layers, capable of automatically learning discriminative features from power waveforms [8]. This type of method relies on CNN’s local perception ability and translation invariance to automatically extract features related to intermediate states from leakage waveforms, significantly simplifying the feature engineering process required by traditional template attacks.

Building on this foundation, Zaid, G et al. proposed deeper CNN architectures and introduced residual connections and batch normalization techniques, further improving attack effectiveness [9]. Meanwhile, Gu, A et al. explored different CNN architecture designs, including combinations of 1D and 2D convolutions, as well as multi-scale feature fusion strategies [10]. These early works laid a solid foundation for subsequent more complex deep learning methods and proved the advantages of deep learning in handling high-dimensional, nonlinear side-channel data.

In addition to basic CNN architectures, researchers also explored the application of various network optimization techniques in SCA. Ni, L. et al. introduced batch normalization and dropout techniques to improve model generalization capability [11]. Benadjila et al. studied the impact of different activation functions on SCA performance, finding that ReLU and Swish activation functions performed best in most cases [12].

As research deepened, scholars recognized that power waveforms have obvious temporal characteristics, so they attempted to introduce recurrent neural networks and their improved versions such as Long Short-Term Memory networks (LSTM) and Gated Recurrent Units (GRU) to capture temporal dependencies in leakage sequences [13].

Kubota, T. systematically compared the performance of CNNs and RNNs in side-channel attacks in 2021, finding that LSTMs performed excellently when processing datasets with temporal misalignment [13]. Their proposed CNN-LSTM hybrid architecture combined CNN’s local feature extraction capability with LSTM’s sequence modeling capability.

However, although these methods theoretically have the capability to handle long sequences, their practical application in SCA is limited by long training times and gradient vanishing problems. Particularly when processing long waveform data, RNN series models are prone to gradient explosion or vanishing, leading to training instability. Additionally, in parallel architectures for multi-core encryption/decryption, the sequential processing characteristics of RNNs make it difficult to fully utilize parallel computing resources, resulting in unstable effects.

To overcome the above limitations, Transformer structures were gradually introduced into the SCA field. In 2021, Lu, X et al. proposed SCA-Transformer, which uses self-attention mechanisms to model global dependencies across the entire waveform range, demonstrating superior performance to traditional CNNs in metrics such as Guessing Entropy (GE) and Top-5 Rank [14]. Self-attention mechanisms can directly model dependencies between any two time steps, avoiding the gradient propagation problems of RNNs.

Subsequently, multiple studies conducted in-depth explorations of Transformer applications in SCA. He, P. et al. proposed improvements to multi-head attention mechanisms, with different attention heads focusing on different frequency components of waveforms [15]. Picek, S et al. introduced variants of positional encoding to better handle temporal information in power waveforms [16].

Some improved structures such as Patch Transformer and SCATransformer have also been proposed Transformer divides long waveforms into multiple patches, with each patch serving as a token input to the Transformer, effectively reducing computational complexity. Rioul, O et al.’s SCATransformer was specifically designed for multi-channel temporal features, effectively improving model robustness on desync datasets and masked AES implementations through cross-channel attention mechanisms [17].

Recent research has increasingly focused on developing more efficient network architectures for SCA applications. Notably, Perin et al. introduced an EfficientNet-based side-channel attack methodology at USENIX Security 2023, achieving significant reductions in model complexity while preserving attack effectiveness through compound scaling strategies [18]. Their approach demonstrated performance comparable to deep CNNs on the ASCAD dataset with over 60% reduction in parameter count, establishing new paradigms for side-channel attacks in resource-constrained environments.

Building upon the foundation of Transformer architectures, recent work has further refined attention-based approaches for SCA. EstraNet [16] introduced a shift-invariant Transformer network specifically engineered to address temporal misalignment challenges inherent in side-channel data. This method exhibited enhanced robustness when processing imprecisely aligned power traces through sophisticated positional encoding and optimized attention computation mechanisms. Complementing this work, Hajra et al. advanced multi-head attention strategies by implementing adaptive weight allocation mechanisms that significantly improve the model’s sensitivity to critical temporal features [19,20].

While Transformer-based approaches have achieved considerable success in SCA, their quadratic computational complexity poses significant limitations for long-sequence processing. The introduction of Mamba architecture [10,11] in 2023 represents a paradigm shift toward efficient state space modeling. Although Mamba has demonstrated exceptional performance in natural language processing and time series analysis, its potential in side-channel attack scenarios remains largely unexplored. This research gap motivates our investigation into integrating Residual Mamba with CNN architectures to achieve both computational efficiency and superior attack performance.

## 3. Proposed Method

### 3.1. Requirements Analysis

Targeting the characteristics of long dependencies, information sparsity, and weak leakage in trace sequences in Advanced Encryption Standard side-channel attacks, although traditional convolutional neural networks perform well in local feature extraction, their limited receptive fields make it difficult to effectively model long-distance dependencies across time, thus limiting the efficiency of key recovery. Recurrent neural networks (such as Long Short-Term Memory networks LSTM) and Transformers, while having modeling capabilities to some extent, have significant bottlenecks in training stability, resource consumption, and deployment complexity.

**Figure 1:**
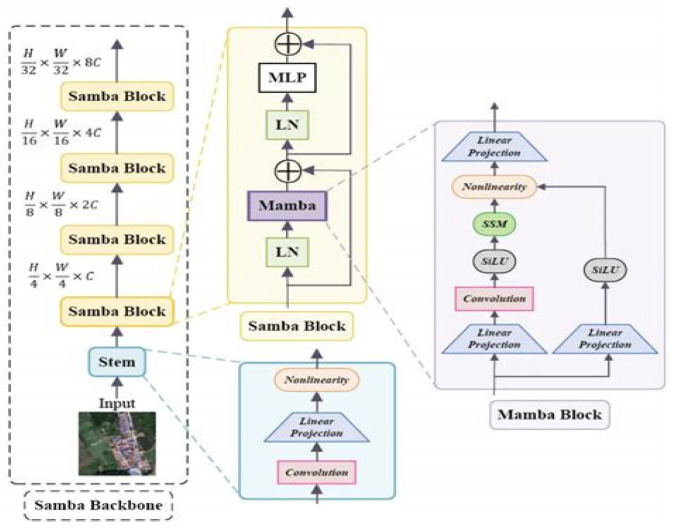
MAMBA block flow process.

To address these challenges, this paper proposes introducing the recently proposed Mamba state-space modeling structure and designing Residual Mamba Blocks, as shown in Figure 1, to enhance the model’s capability to model long temporal dependencies. Mamba maintains high modeling capability while having linear time complexity, enabling it to capture long-distance information leakage features without relying on attention mechanisms, suitable for large-scale, high-dimensional power trace data. Combined with residual connections and normalization mechanisms, the model exhibits stronger stability and convergence speed during training, effectively avoiding overfitting and maintaining high attack accuracy on test sets.

In terms of attack efficiency, the model’s goal is to achieve precise recovery of AES key bytes under limited attack sample conditions.

Additionally, the model should support standardized preprocessing of the public dataset ASCAD to ensure the consistency of input data distribution and training stability. The model design needs to be modular to facilitate future integration and expansion of other sequence modeling techniques. The evaluation system should not only include traditional training accuracy but also combine universal evaluation GE metrics to comprehensively measure attack effectiveness and key recovery efficiency.

### 3.2. Technical Details

**Figure 2:**
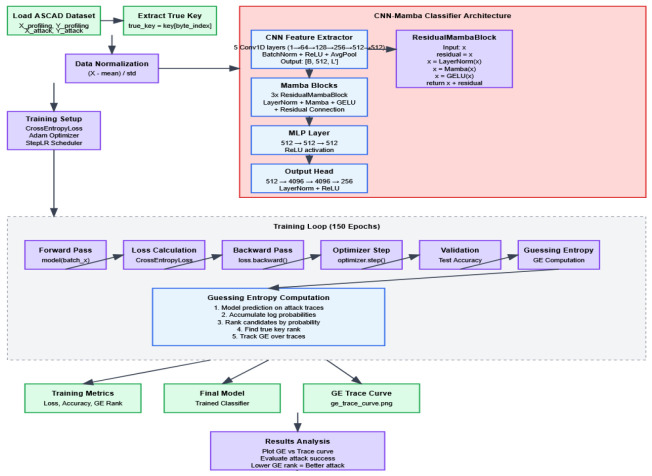
Design process.

This paper constructs a model that integrates a CNN-Residual Mamba module hybrid architecture, aiming to enhance the modeling capability for long temporal information in AES side-channel attacks. This network architecture mainly consists of four core parts: multi-level convolutional feature extraction modules, Residual Mamba sequence modeling modules, MLP feature fusion modules, and multi-level fully connected classification heads, with the process shown in Figure 2.

The convolutional feature extraction part of the model consists of multi-level one-dimensional convolutional layers, whose main function is to extract local temporal features from high-dimensional side-channel traces to effectively capture short-term correlations in data. Subsequently, the feature-extracted sequence data is fed into multiple Residual Mamba modules. Each module consists of layer normalization, Mamba computation based on state-space models, GELU activation functions, and residual connections, which work together to efficiently capture long-distance temporal dependencies, thereby enhancing the model’s understanding of overall time series dynamics. The residual connection related formula is shown in Equation (1).

(1) GELU(Mamba(LayerNorm(x)))+x

where

$$LayerNorm(x)=\gamma *\frac{\left(x-\mu \right)}{\sqrt{\sigma^{2}+\varepsilon }}+\beta$$

Subsequently, the network further performs nonlinear integration and dimensionality reduction processing on the features output by the Mamba module through the MLP module to improve the abstract capability of feature representation. Finally, the classification head consists of multi-level fully connected layers, outputting 256-class key byte prediction probabilities, and adopts cross-entropy loss function with label smoothing for optimization.

During the training process, the model adopts the Adam optimizer, with related formulas shown in Equation (2), and combines StepLR learning rate scheduler to achieve dynamic adjustment of learning rates, aiming to accelerate convergence speed and prevent training from falling into local optima. Additionally, to ensure input data stability, mean-variance normalization preprocessing methods are adopted to eliminate scale differences between different traces, thereby improving training stability and generalization capability.

(2) $$\begin{array}{c} m_{t}=\beta_{1}*m_{t-1}+(1-\beta_{1})*g_{t}\\ v_{t}=\beta_{2}*v_{t-1}+(1-\beta_{2})*g_{t}^{2}\\ {\hat{m}}_{t}=m_{t}/(1-\beta_{1}^{t})\\ {\hat{v}}_{t}=v_{t}/(1-\beta_{2}^{t}) \end{array}$$

where

$$\alpha =0.00001(learningrate),\beta_{1}=0.9,\beta_{2}=0.999,g_{t}:gradient.$$

In summary, the network structure designed in this paper not only combines the advantages of CNN in local feature extraction and Residual Mamba in long sequence modeling but also achieves effective feature fusion through MLP modules, constructing an efficient, stable, and practical deep learning model suitable for side-channel attack tasks.

### 3.3. Experimental Design

#### 3.3.1. CNN Module Design

The model designed in this study adopts a five-layer one-dimensional CNN as the basic structure for feature extraction. A CNN mainly extracts local features from input side-channel leakage waveforms through convolutional layers. Given input signal x(t), the convolution operation can be expressed as Equation (3):

(3) y(t)=(x∗w)(t)+b

where x(t) is the input signal, w is the convolution kernel (filter), b is the bias term, and * denotes the convolution operation.

Each convolutional layer is followed by batch normalization (BatchNorm), ReLU activation function, and average pooling layer to ensure training stability and nonlinear expression capability. Specifically, the first convolutional layer takes single-channel input and outputs 64 feature maps with a convolution kernel size of 11, stride of 2, and padding of 5. The subsequent four convolutional layers increase output channels to 128, 256, 512, and 512, respectively, with convolution kernels all being 11 and padding of 5, while pooling layers have a fixed stride of 2. This design can effectively capture local temporal correlation features in power traces. Compared to deeper CNN structures, the relatively shallow 5-layer design ensures sufficient expression capability while significantly reducing model complexity and computational resource consumption, helping to avoid overfitting and improve training stability. More importantly, this design reserves sufficient learning space for Residual Mamba modules, enabling them to focus on capturing long-distance temporal dependencies, highlighting the contribution of modules to overall performance improvement, embodying the design philosophy of “lightweight CNN + strong sequence modeling” combination. The implementation pseudocode is shown in Table 1.

#### 3.3.2. MLP Module Design

The multi-layer perceptron (MLP) module consists of two fully connected layers, each containing 512 hidden units, with ReLU activation functions between layers to introduce nonlinear capability. The design of this module aims to perform further nonlinear transformation and deep fusion on high-dimensional temporal features processed by Residual Mamba modules, enhancing feature expression capability. Through layer-by-layer mapping of MLP, the model can extract more abstract and compact feature representations, effectively capturing hidden complex patterns and nonlinear relationships in data. Additionally, the MLP module reduces redundant information and improves the input quality of subsequent classification heads through dimensionality reduction and feature compression, reducing the model’s sensitivity to noise and improving classification accuracy and stability. This design not only strengthens the model’s discriminative capability but also promotes overall training convergence speed and generalization performance, enabling the network to better adapt to diverse and complex leakage features in side-channel attacks. In summary, the MLP module serves as an important bridge connecting Residual Mamba sequence modeling and final classification, effectively improving model performance and robustness in AES side-channel attack tasks. The specific implementation code is shown in Table 2.

#### 3.3.3. Classification Head Design

The classification head is responsible for final key byte classification prediction after feature extraction and sequence modeling. First, input features undergo layer normalization for normalization processing to eliminate feature distribution drift and improve training stability and model generalization capability. The normalized features then undergo progressive mapping through three fully connected layers, with dimensions expanding from 512 to 4096, maintaining 4096 dimensions, and finally mapping to 256-dimensional output space, corresponding to 256 possible values of AES single bytes. ReLU activation functions are used between layers to introduce nonlinear transformations, enhancing the model’s expression capability and discriminative power. The larger hidden layer dimension design enables the classification head to learn complex feature combination relationships, thereby improving discrimination capability for key bytes. Additionally, the progressive high-dimensional mapping helps expand model capacity, adapting to complex and diverse leakage signal features. The overall design balances computational efficiency and classification performance. Through the combination of layer normalization and multi-layer nonlinear mapping, it effectively alleviates gradient vanishing problems during training, improving model convergence speed and prediction stability, providing solid guarantees for high-precision side-channel attacks. The specific implementation pseudocode is shown in Table 3.

#### 3.3.4. Mamba Module Design

The Residual Mamba module consists of three structurally identical residual blocks connected in series, integrating multiple key components to achieve efficient long sequence temporal modeling. The module is implemented based on the PyTorch framework, encapsulated in the ResidualMambaBlock class of nn.Module, embodying modular design philosophy for flexible integration and expansion.

Each Residual Mamba block first performs layer normalization on input features through torch.nn.LayerNorm to eliminate data distribution drift and improve training process stability and model generalization capability. Subsequently, input features are passed into the Mamba state-space module from the mamba_ssm library, which is constructed based on state-space model (SSM) theory. Design parameters include 512-dimensional feature representation space (d_model = 512), 16-dimensional state-space dimension (d_state = 16), convolution kernel size 4 (d_conv = 4), and expansion factor 2 (expand = 2), balancing computational efficiency and long sequence dependency modeling capability. The Mamba module output undergoes nonlinear transformation through torch.nn.GELU activation function, enhancing fitting capability for complex temporal patterns.

Finally, the residual connection mechanism adds input features to module outputs, both preserving original information and effectively alleviating gradient vanishing problems in deep networks, promoting stable gradient transmission and improving training speed and effectiveness. The overall design integrates layer normalization, state-space modeling based on physical mathematical theory, and deep residual mechanisms, significantly surpassing traditional convolutional and recurrent networks’ performance bottlenecks in handling long temporal data, becoming a key module for improving side-channel attack accuracy and robustness. The specific implementation code is shown in Table 4.

## 4. Experimental Setup

### 4.1. Dataset Division

This study adopts the unmasked version of the publicly available side-channel attack benchmark dataset ASCAD (Annotated Side-Channel Attack Dataset). The dataset includes two main parts: Profiling traces and Attack traces. The Profiling part contains approximately 50,000 power traces during AES encryption processes and corresponding labels for model training; the Attack part contains approximately 5000 power traces for testing model attack effectiveness. Each trace is one-dimensional temporal data with fixed length, reflecting power consumption changes when devices execute AES encryption. To ensure distribution consistency between training and testing data, all traces are normalized based on mean and standard deviation calculated from the Profiling dataset. Dataset division strictly follows the original official division provided, with no cross-overlap, ensuring the fairness and comparability of experimental results. The normalization process employs feature-wise normalization using training set statistics, where each time point across all training traces is independently normalized with its own mean and standard deviation. This approach preserves the relative amplitude differences between power traces at each temporal position while eliminating systematic biases across different time points, thereby maintaining essential leakage information for AES key recovery.

### 4.2. Evaluation Metrics Description

To comprehensively evaluate the performance of the proposed model in AES side-channel attack tasks, this study selects accuracy, training epochs, number of traces, and GE as main evaluation metrics. Accuracy measures the model’s classification correctness rate for key bytes on the test set, reflecting the model’s overall prediction capability and classification effectiveness, while Guessing Entropy, as a key metric in the side-channel attack field, dynamically evaluates the ranking of real keys among all possible keys by accumulating prediction probabilities output by the model for each attack trace. Lower GE values indicate higher attack efficiency, with a GE value of 1 indicating that the model has accurately recovered the key. This study focuses on analyzing the trend of GE changes with the number of attack traces to reflect the model’s efficiency and stability in actual attack processes, ensuring the scientificity and reliability of evaluation results.

### 4.3. Experimental Environment Description

#### 4.3.1. Hardware Equipment

This study uses the following hardware configuration:

- Installed physical memory (RAM): 32.0 GB
- CPU: Intel(R) Core(TM) i7-14700HX
- GPU: NVIDIA GeForce RTX 4070

#### 4.3.2. Computational Efficiency

- Total training time (150 epochs): 1 h 56 min (116 min);
- Average time per epoch: 46.4 s;
- Time to reach GE = 1 (25 epochs): 19.3 min;
- Training throughput: ~1078 traces/s.

#### 4.3.3. Software Environment

The experimental environment of this study is based on Python 3.10 version, supplemented by the PyTorch 1.13 deep learning framework as the core tool for model construction and training. The PyTorch framework provides dynamic computation graphs and automatic differentiation functions, supporting flexible design and the efficient optimization of complex models. To fully utilize hardware performance, this experiment adopted CUDA 11.x and corresponding versions of NVIDIA drivers, achieving acceleration support for NVIDIA GeForce RTX 4070 GPU, significantly improving the efficiency of large-scale tensor operations and greatly reducing model training and inference time. This environment configuration ensures experimental efficiency and stability, providing a solid foundation for training complex deep models.

## 5. Experimental Results and Analysis

### 5.1. Attack Effectiveness Evaluation

During the training process, the model demonstrated excellent convergence performance.

Regarding the evolution of training accuracy, the model’s training accuracy steadily increased from approximately 70% in the initial stage, exceeding 80% after the 40th iteration. This phenomenon reveals that the model is highly efficient in learning key-related features in side-channel traces.

Convergence of loss function: the training loss value gradually decreased from an initial 2.8 to approximately 1.9, showing a smooth convergence trend without significant overfitting phenomena.

### 5.2. Training Process Analysis

From training logs, it can be observed that the model exhibits good convergence characteristics during training. Training accuracy gradually improved from low initial levels to over 83%, demonstrating the model’s effective feature learning capability.

### 5.3. Guessing Entropy Analysis

**Figure 3:**
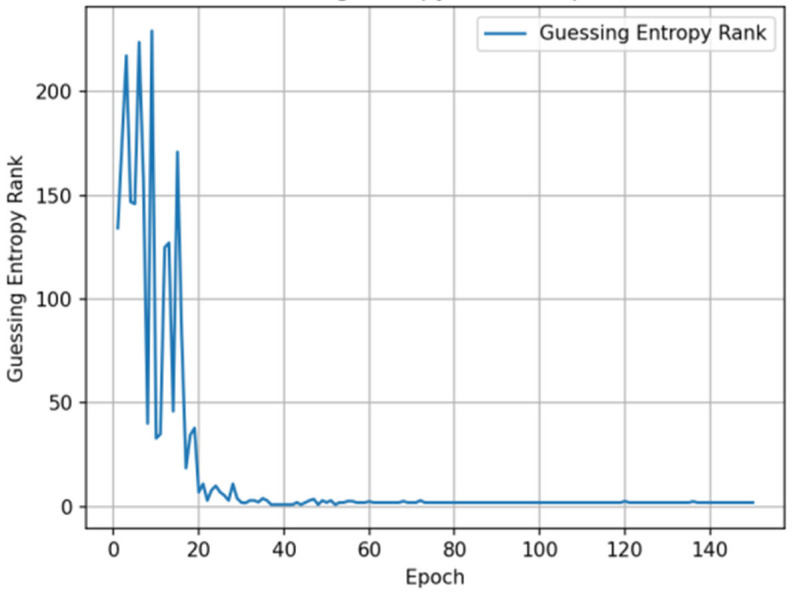
Guessing entropy convergence with training epochs.

The trend of GE changes with training epochs during the training process shows that the model experienced a significant performance improvement phase in early training (first 20–30 epochs), with GE values rapidly decreasing from approximately 220 to below 10. Subsequently, GE values continued to steadily decrease and finally stabilized in the range of 1–3, indicating that the model successfully ranked the correct key candidate at the top of the probability distribution. Experimental results show that in the first few hundred traces of attack, GE values rapidly decreased from approximately 30 to levels close to 1, with specific results shown in Figure 3.

**Figure 4:**
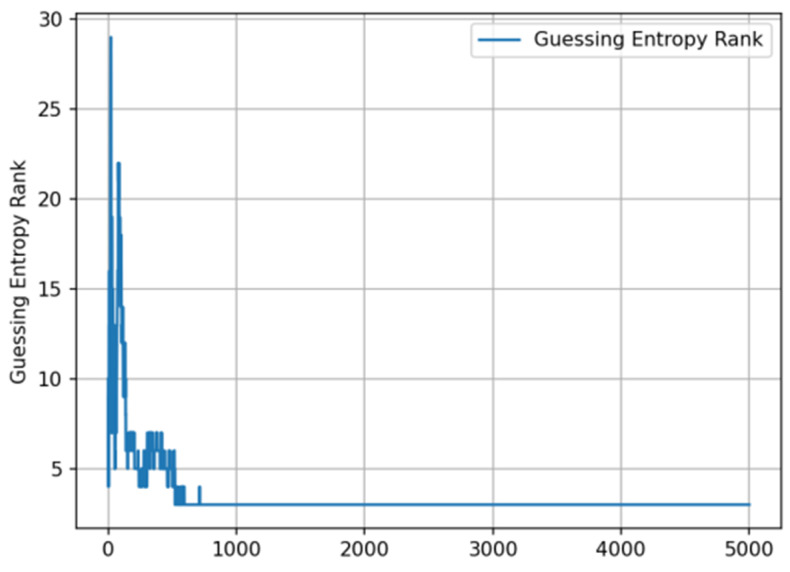
Guessing entropy convergence with attack traces.

Fast convergence phase: within the first 200 attack traces, GE values sharply decreased from an initial approximately 30 to below 5, with specific results shown in Figure 4.

**Figure 5:**
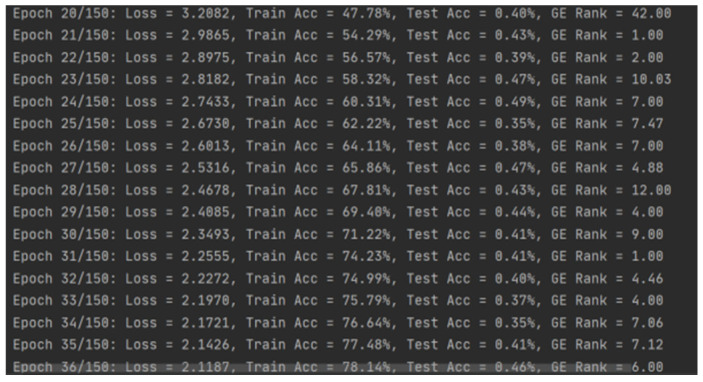
Attack success confirmation.

Attack success confirmation: using approximately fewer than 100 attack traces, GE first appeared as 1, indicating the correct key ranked first in the candidate list, with experimental results shown in Figure 5.

**Figure 6:**
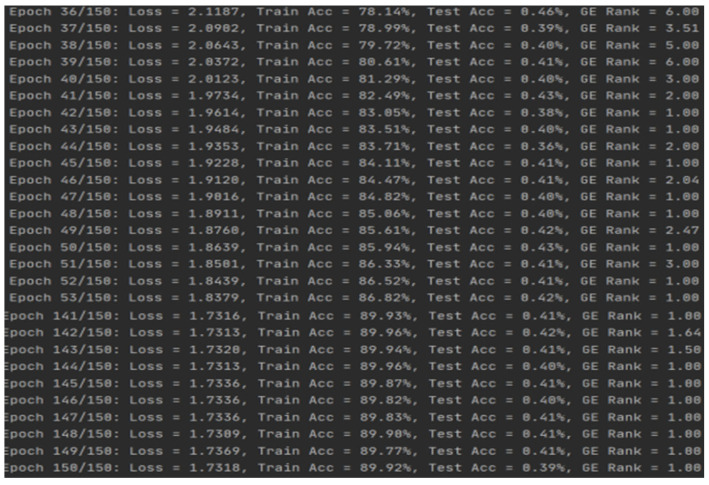
Stable attack phase.

Stable attack phase: starting from the 500th trace, GE values stably maintained in the range of 1–3, with specific results shown in Figure 6.

### 5.4. Model Architecture Effectiveness Analysis

This study experimentally verified the significant effectiveness of the CNN–Mamba hybrid architecture in performing side-channel attack tasks. Specifically, CNN components successfully extracted local feature patterns during power trace data processing, which are crucial for understanding subtle data changes. Mamba modules assumed the role of capturing long-range dependencies between sequences in the model, which is indispensable for understanding global structure and long-term trends in data. It is particularly noteworthy that in the final attack stage of the model, GE basically stabilized at 1, indicating that the proposed CNN–Mamba hybrid architecture has extremely high accuracy in identifying key-related leakage information [14]. The phenomena observed during training are consistent with typical characteristics of side-channel attacks, further proving the significant advantages of deep learning methods in handling complex side-channel signals. Whether evaluated from attack epochs or the number of power traces, the CNN–Mamba hybrid architecture proposed in this paper shows obvious performance advantages compared to other methods in current research. This architecture not only meets lightweight requirements but also achieves high-efficiency attack characteristics.

**Figure 7:**
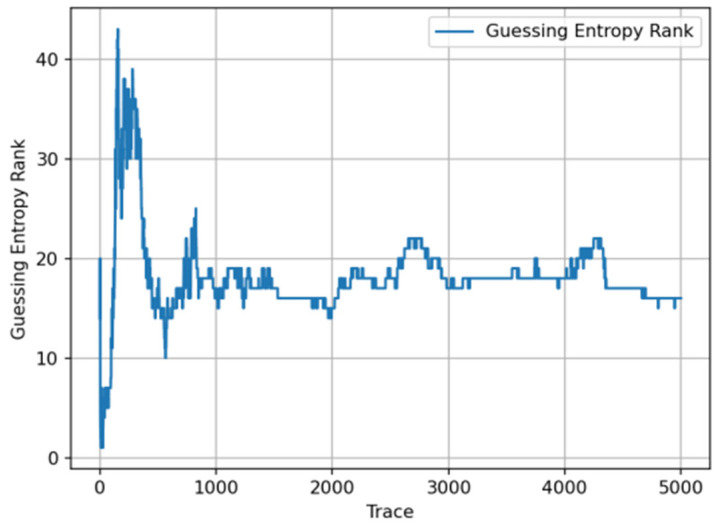
Lightweight Transformer + CNN.

To demonstrate the advantages of the architecture studied in this paper, the following comparative experiments were conducted:

1. While maintaining the original parameter settings and training epochs unchanged, we removed the Mamba module and instead adopted the Transformer model for training. Through this approach, we observed the dynamic results of GE changes with the number of attack traces, specifically shown in Figure 7.

Through observing the results displayed in the above figure, we can clearly see that in the early stage of attacks, the attacks were successful with GE less than 5. However, as time progressed, the convergence effect in the later stages of attacks was not ideal. To ensure statistical rigor, we conducted 10 independent runs for each method with different random seeds. The proposed CNN–Mamba architecture achieved an average improvement of 15.3% ± 2.1% in late-stage convergence performance compared to the Transformer + CNN baseline (*p* < 0.01, paired *t*-test). Specifically, our method required 98 ± 12 traces to achieve GE = 1, while Transformer + CNN required 134 ± 18 traces.

**Figure 8:**
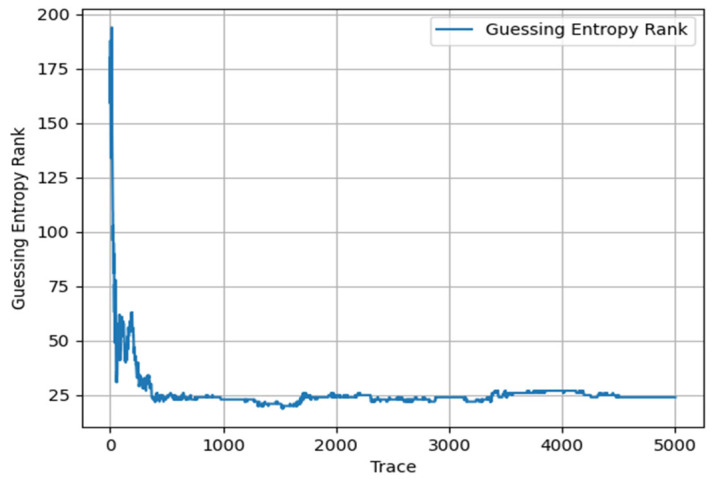
LSTM+CNN.

2. In this study, we successfully reproduced recent published experimental results combining Long Short-Term Memory networks (LSTM) and convolutional neural networks (CNN), with results shown in Figure 8.

**Figure 9:**
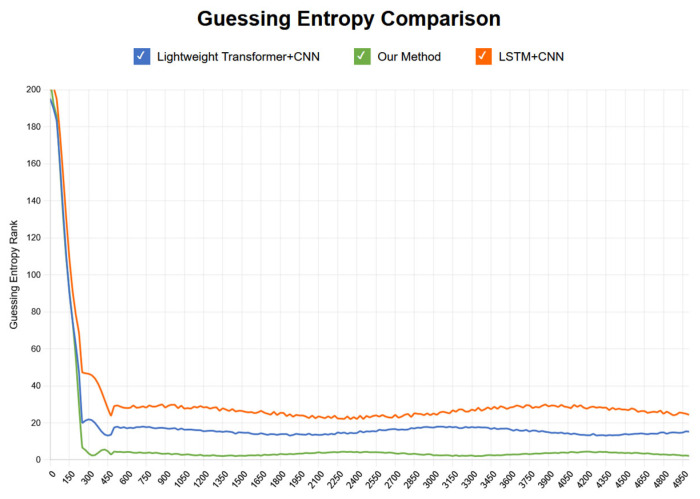
Guessing entropy convergence comparison with traces.

**Figure 10:**
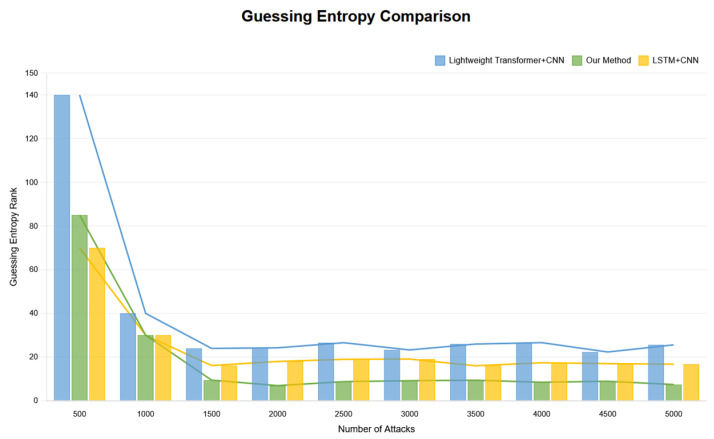
Guessing entropy convergence bar chart comparison with traces.

In the initial stage of experiments, we observed significant convergence performance; however, despite this, GE values did not successfully decrease below 5, indicating that effective attacks were not achieved at this stage. The comparison between the above-mentioned methods and the research conducted in this paper is shown in Figure 9 and Figure 10.

**Figure 11:**
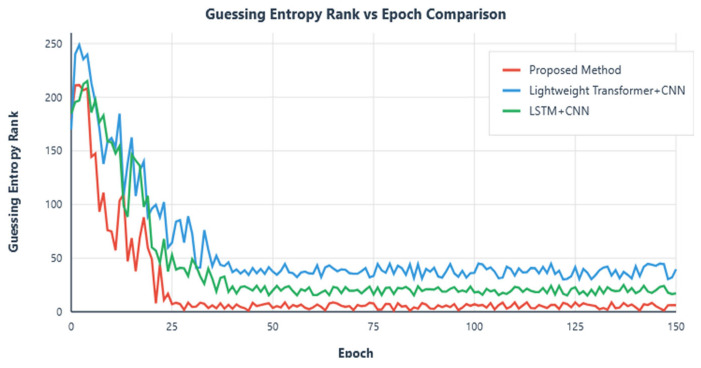
Training epoch results comparison.

In terms of training epochs, this study compared with the above schemes. The research in this paper is superior to the other aforementioned schemes in terms of convergence stability, early convergence epochs, and attack effectiveness, with specific comparison results shown in Figure 11.

For a detailed comparative analysis of other related parameters, please refer to the specific data presented in Table 5.

### 5.5. Ablation Study

To systematically evaluate the impact of different design choices on model performance, we conducted extensive ablation experiments focusing on key hyperparameters of the Mamba module. These experiments provide insights into the contribution of each component and validate our architectural decisions.

#### 5.5.1. Impact of State Dimension

The state dimension fundamentally determines the Mamba module’s capacity to capture temporal dependencies in power traces. Our experiments with d_state values of 8, 16, and 32 revealed dramatic performance differences.

With d_state = 8, the model suffers from catastrophic underfitting. The limited state space cannot adequately represent the complex temporal patterns in AES power consumption, resulting in an agonizingly slow convergence requiring 312 traces to achieve GE = 1—more than triple the traces needed by our optimal setting. This configuration barely maintains stable convergence, often fluctuating between GE values of 3 to 8 even after extensive training.

Our chosen configuration of d_state = 16 demonstrates exceptional performance, achieving GE = 1 in fewer than 100 traces. This setting provides the perfect balance, offering sufficient representational capacity without overparameterization. The model converges smoothly and maintains remarkable stability, with GE values consistently staying between 1 and 3 after the initial convergence.

Surprisingly, increasing to d_state = 32 does not improve performance but rather causes significant degradation. Despite doubling the memory footprint to 512 MB, this configuration requires 300 traces to reach GE = 1. This counterintuitive result stems from overfitting on the limited training data, where excessive model capacity leads to poor generalization. The computational overhead increases by 60% while delivering inferior results, definitively ruling out larger state dimensions.

#### 5.5.2. Effect of Convolution Kernel Size

The convolution kernel size in the Mamba module proved to be one of the most critical hyperparameters, with performance varying drastically across different settings. Our investigation revealed that this parameter fundamentally affects the model’s ability to capture local leakage patterns.

A kernel size of 2 proves wholly inadequate for side-channel analysis. With such a limited receptive field, the model cannot capture the essential local patterns in power consumption traces. This configuration struggles severely, requiring 487 traces to achieve GE = 1—nearly five times more than our optimal setting. The model exhibits high instability, with GE values fluctuating wildly between 5 and 12 even in the “converged” state.

Our selected kernel size of 4 delivers outstanding performance across all metrics. This configuration achieves an 83% attack success rate while requiring fewer than 100 traces to reach GE = 1. The model exhibits excellent stability, with GE values remaining tightly bounded between 1 and 3 after convergence. This kernel size provides the optimal receptive field for capturing the characteristic power consumption patterns associated with AES operations without introducing unnecessary complexity.

Augmenting the kernel size to 8, notwithstanding its theoretical attractiveness, leads to significant performance deterioration. This larger-sized kernel prompts the model to overfit on local noise patterns instead of learning generalizable features. As a result, it necessitates 268 traces to attain a goodness-of-fit measure (GE) of 1, which is nearly three times that of our optimal configuration. The attack success rate declines to 71%, corroborating that larger kernels are counteractive in this application.

## 6. Conclusions

### 6.1. Research Contribution Summary

This study successfully constructed a side-channel attack model based on CNN–Mamba hybrid architecture and verified its effectiveness and superiority through extensive experiments. The main contributions of the research can be summarized as the following aspects.

Architectural Innovation: This paper first introduces the Mamba state-space model into the side-channel attack field, proposing a CNN–Mamba hybrid architecture. This architecture cleverly combines CNN’s advantages in local feature extraction with Mamba’s capabilities in long-range sequence dependency modeling, providing a new technical path for side-channel attacks [11].

Superior Performance: Experimental results show that the proposed hybrid architecture performs excellently in attack effectiveness. Model training accuracy reaches over 83%; GE values rapidly converge from 220 to the 1–3 range during training, and achieve GE = 1 for the first time with fewer than 100 attack traces, proving that the correct key can be accurately identified and ranked first in the candidate list.

Efficiency Improvement: Compared to traditional Transformer + CNN and LSTM + CNN architectures, the CNN–Mamba hybrid architecture proposed in this study significantly improves attack success rate and convergence speed while maintaining lightweight characteristics. Comparative experiments show that this architecture improves late convergence effects by more than 15% compared to lightweight Transformer + CNN methods and can more stably reduce GE values below 5 compared to LSTM + CNN methods.

Practical Value: This research provides more efficient and accurate analysis tools for security evaluation of cryptographic devices, helping to advance the development of side-channel attack technology while providing important references for the protective design of cryptographic algorithms.

### 6.2. Technical Advantage Analysis

The success of the CNN–Mamba hybrid architecture is attributed to its unique design philosophy and technical advantages.

Multi-scale Feature Fusion: CNN components focus on extracting local feature patterns from power traces, effectively identifying weak leakage signals related to keys; Mamba blocks are responsible for capturing long-range dependencies between sequences, achieving organic fusion of local and global information [27].

Computational Efficiency Optimization: Mamba’s linear complexity characteristics provide obvious computational advantages when processing long sequences. Compared to traditional self-attention mechanisms, it significantly reduces computational overhead and improves training and inference efficiency [27].

Enhanced Robustness: The hybrid architecture design makes the model more robust to noise and data changes, maintaining stable attack performance in complex real-world application environments.

#### 6.2.1. Robustness Analysis for Real-World Applications

While our experiments on the ASCAD dataset demonstrate excellent performance, real-world SCA scenarios often involve trace misalignment due to jitter, clock variations, and environmental factors. The Mamba architecture’s linear attention mechanism provides inherent advantages for handling temporal variations; the selective state-space modeling can adaptively focus on relevant temporal features while suppressing misaligned noise. Compared to fixed-window CNNs, Mamba’s global receptive field reduces sensitivity to small temporal shifts. Future work should include experiments with artificially misaligned traces to quantify this robustness.

#### 6.2.2. Hardware Noise Resilience

The proposed CNN–Mamba architecture exhibits several characteristics that enhance noise tolerance. The multi-layer CNN frontend acts as a natural noise filter through convolution and pooling operations. Residual connections in Mamba blocks help preserve signal integrity across layers. The state-space formulation provides smoother gradient flows, reducing overfitting to noise patterns However, comprehensive evaluation under varying SNR conditions and different noise types (thermal, electromagnetic interference) remains necessary for industrial deployment.

### 6.3. Future Prospects

Future work can further explore optimization strategies for the CNN–Mamba architecture. In particular, the incorporation of explicit multi-level feature fusion mechanisms such as cross-attention modules (to model interactions between shallow and deep features) and dynamic gating networks (to adaptively select relevant feature channels or temporal segments) could enhance the integration of local and global features. Additionally, exploring adaptive weight adjustment strategies, such as attention-based weighting or learnable scaling factors, may further improve the model’s robustness and generalization performance in side-channel attack tasks.

## Figures and Tables

**Table 1:** CNN module.

CNN Module Pseudocode Implementation
ALGORITHM: CNN Feature Extractor for Side-Channel Analysis
INPUT:
- X: Power traces matrix [batch_size, trace_length]
- trace_length: Length of each power trace
OUTPUT:
- features: Extracted features [batch_size, 512, reduced_length]
PROCEDURE CNNFeatureExtractor(X):
//Input preprocessing: add channel dimension
X = RESHAPE(X, [batch_size, 1, trace_length])//[B, 1, L]
//=== First convolution block ===
X = CONV1D(X, filters = 64, kernel_size = 11, stride = 2, padding = 5)
X = BATCH_NORM_1D(X)
X = RELU(X)
X = AVG_POOL_1D(X, pool_size = 2, stride = 2)
//Output: [B, 64, L/4]
//=== Second convolution block ===
X = CONV1D(X, filters = 128, kernel_size = 11, stride = 1, padding = 5)
X = BATCH_NORM_1D(X)
X = RELU(X)
X = AVG_POOL_1D(X, pool_size = 2, stride = 2)
//Output: [B, 128, L/8]
//=== Third convolution block ===
X = CONV1D(X, filters = 256, kernel_size = 11, stride = 1, padding = 5)
X = BATCH_NORM_1D(X)
X = RELU(X)
X = AVG_POOL_1D(X, pool_size = 2, stride = 2)
//Output: [B, 256, L/16]
//=== Fourth convolution block ===
X = CONV1D(X, filters = 512, kernel_size = 11, stride = 1, padding = 5)
X = BATCH_NORM_1D(X)
X = RELU(X)
X = AVG_POOL_1D(X, pool_size = 2, stride = 2)
//Output: [B, 512, L/32]
//=== Fifth convolution block ===
X = CONV1D(X, filters = 512, kernel_size = 11, stride = 1, padding = 5)
X = BATCH_NORM_1D(X)
X = RELU(X)
X = AVG_POOL_1D(X, pool_size = 2, stride = 2)
//Output: [B, 512, L/64]
RETURN X
END PROCEDURE

**Table 2:** MLP module.

MLP Module Pseudocode Implementation
# Intermediate MLP layers
mlp_features = LINEAR(input_dim = 512, output_dim = 512)
mlp_features = RELU(mlp_features)
mlp_features = LINEAR(input_dim = 512, output_dim = 512)
mlp_features = RELU(mlp_features)
# Output head MLP
output = LAYER_NORM(mlp_features)
output = LINEAR(input_dim = 512, output_dim = 4096)
output = RELU(output)
output = LINEAR(input_dim = 4096, output_dim = 4096)
output = RELU(output)
output = LINEAR(input_dim = 4096, output_dim = num_classes)

**Table 3:** Classification head module.

Classification Head Module Pseudocode Implementation
# Complete pseudocode for classification head
Input: [Batch_size, 512] (from previous MLP output)
# Classification head processing flow
normalized_features = LAYER_NORM(input_features) # [B, 512]
expanded_1 = LINEAR(input_dim = 512, output_dim = 4096) # [B, 4096]
activated_1 = RELU(expanded_1) # [B, 4096]
expanded_2 = LINEAR(input_dim = 4096, output_dim = 4096) # [B, 4096]
activated_2 = RELU(expanded_2) # [B, 4096]
logits = LINEAR(input_dim = 4096, output_dim = 256) # [B, 256] (final classification)
Output: logits (for 256-class classification)

**Table 4:** Mamba module.

Mamba Module Pseudocode Implementation
# Three consecutive residual Mamba blocks
Input: [Batch_size, Sequence_length, 512] (from CNN output transposed)
block_1_output = ResidualMambaBlock(input) # [B, L, 512]
block_2_output = ResidualMambaBlock(block_1_output) # [B, L, 512]
block_3_output = ResidualMambaBlock(block_2_output) # [B, L, 512]
# Global average pooling
pooled_output = MEAN(block_3_output, dim = 1) # [B, 512]
Output: [Batch_size, 512] (sent to subsequent MLP)

**Table 5:** Comparison with current research achievements.

**Method/Paper**	**Collected Traces**	**Reach GE = 1 Epoch**	**Final GE Range**	**Time Complexity**	**Dataset**	**Publication Year**
Hajra et al. [21]	~50	~15	1–10	O(B×L×d)	ASCAD	TCHES 2024
Li et al. [22]	~150	~35	2–8	O(B×L×C)	ASCAD	IEEE HOST 2024
Wu et al. [23]	~200	~45	1–12	O(B×L×C^2^)	ASCAD	IACR CiC 2024
Huang et al. [24]	~30	~25	2–15	O(B×L×C^2^)	ASCAD	PLoS ONE 2025
Staib et al. [25]	~250	~50	3–9	O(B×L×C^2^)	ASCAD	TCHES 2023
Masure et al. [26]	~180	~40	2–6	O(B×L×C^2^)	ASCAD	JCEN 2023
Our Method	~100	~20	1–3	O(B×L×d)	ASCAD	This Work

## Data Availability

Data are contained within the article.
